# Metagenomic survey reveals global distribution and evolution of microbial sialic acid catabolism

**DOI:** 10.3389/fmicb.2023.1267152

**Published:** 2023-09-29

**Authors:** Yisong Li, Yeshun Fan, Xiaofang Ma, Ying Wang, Jie Liu

**Affiliations:** ^1^School of Public Health, Qingdao University, Qingdao, China; ^2^Qingdao Municipal Center for Disease Control and Prevention, Qingdao, China

**Keywords:** sialic acid catabolism, metagenomics, gene distribution, evolutionary mechanism, horizontal gene transfer

## Abstract

Sialic acids comprise a varied group of nine-carbon amino sugars found mostly in humans and other higher metazoans, playing major roles in cell interactions with external environments as well as other cells. Microbial sialic acid catabolism (SAC) has long been considered a virulence determinant, and appears to be mainly the purview of pathogenic and commensal bacterial species associated with eukaryotic hosts. Here, we used 2,521 (pre-)assembled metagenomes to evaluate the distribution of SAC in microbial communities from diverse ecosystems and human body parts. Our results demonstrated that microorganisms possessing SAC globally existed in non-host associated environments, although much less frequently than in mammal hosts. We also showed that the ecological significance and taxonomic diversity of microbial SAC have so far been largely underestimated. Phylogenetic analysis revealed a strong signal of horizontal gene transfer among distinct taxa and habitats, and also suggested a specific ecological pressure and a relatively independent evolution history in environmental communities. Our study expanded the known diversity of microbial SAC, and has provided the backbone for further studies on its ecological roles and potential pathogenesis.

## Introduction

Sialic acids, a structurally diverse family of nine-carbon amino sugars, are present in humans and other metazoans constituting glycan chains of glycoconjugates (Angata and Varki, 2002; Vimr et al., 2004). This positional feature contributes a major role in modifying cell surfaces and mediating a range of cell–cell or cell-molecule interactions in eukaryotes (Varki et al., 2011). For pathogenic bacteria, besides exploiting sialic acids for the evasion or recognition of entry into the host, sialic acid catabolism (SAC) could also provide benefits to compete for the restricted source of nutrients in the host (Severi et al., 2007; Kim and Kim, 2023). Thus, microbial SAC has long been considered a virulence determinant in a range of infectious diseases, mainly confining to commensals and pathogens (Almagro-Moreno and Boyd, 2010; Haines-Menges et al., 2015; McDonald et al., 2016).

A complete catabolism of sialic acids was composed of a sialidase (mainly NanH) to release the monosaccharide from the glycan, a transporter system to take up free sialic acids inside the cell, and a suite of intracellular enzymes to convert sialic acids into fructose-6-phosphate that can be catabolized in the central carbon metabolism (Vimr et al., 2004; Vimr, 2013). In general, the most common and well-conserved catabolic pathway (*Escherichia coli* paradigm) is composed of a sequential action of Neu5Ac lyase (NanA), N-acetyl-mannosamine kinase (NanK), and N-acetyl-mannosamine epimerase (NanE) (Plumbridge and Vimr, 1999), although an alternative pathway (*Bacteroidetes* paradigm pathway) has also been identified in certain individual species (Brigham et al., 2009; Roy et al., 2010). Thus, a complete SAC system is usually defined as one that minimally includes orthologues of genes encoding NanA, NanK, and NanE. These three genes usually group together, forming a gene cluster denominated as *nan* (Almagro-Moreno and Boyd, 2009). Our recent analysis further demonstrated that to a large extent, *nanE* may represent the *nan* gene cluster in actinobacteria (Li and Huang, 2022). It should be mentioned that although many common commensals and pathogens may contain genes for only part of the entire pathway, they could still in some way play their roles in SAC within the *in vivo* microbial community (Coker et al., 2021).

A review of sialic acids catabolism by pathogens all over the human body has highlighted the importance of sialic acid as a carbon source for pathogens and the association of SAC with pathogenesis (Haines-Menges et al., 2015). However, only limited research has recognized its distribution in non-mammal-host habitats. Recently, by using a mass spectrometric all-ion-reaction scanning approach, Kleikamp et al. (2020) have revealed an unexpectedly wide distribution and chemical diversity of sialic acids among nonpathogenic environmental bacteria. In accordance with this, experimental and bioinformatic studies have shown that many environmental (non-host-associated) microorganisms could also utilize sialic acids as a nutrient. For example, soil-derived *Corynebacterium glutamicum* isolates could grow on Neu5Ac medium (Gruteser et al., 2012), and this is also true for some free-living *Streptomyces* species (Li et al., 2019; Wang et al., 2022). In addition, Tomás-Martínez et al. have shown that both *Clostridium* and *Chryseobacterium* strains can take up free sialic acids and utilize them as a nutrient, further suggesting the important role of microbial SAC in environmental communities lacking eukaryotic hosts (Tomás-Martínez et al., 2022). Our recent genome-based survey also demonstrated that ~40% of the SAC-positive actinobacterial species have a free-living lifestyle (Li and Huang, 2022). To our knowledge, systematic research on microbial SAC communities in different environments remains limited.

With the development of high-throughput sequencing techniques and the advent of metagenomics, a proliferation of publicly available DNA sequence data from microbial communities residing in diverse ecosystems makes it possible to perform an extensive investigation of gene distribution (Podar et al., 2015; Holert et al., 2018; Viljakainen and Hug, 2021). This culture-independent analysis could also detect genes from organisms that currently are not culturable (Dick et al., 2009). In this study, we screened >2,500 publicly available (pre-)assembled microbial metagenomes to evaluate microbial SAC prevalence, distribution, and taxonomy in natural and engineered environments and in different human body parts. Our analyses expanded the known understanding of the diversity of SAC, and also provided implications for its wide distribution and evolutionary mechanism.

## Results

### Ecological distribution of *nanE* and SAC clusters

A total of 2,521 publicly accessible (pre-)assembled shotgun metagenomes from geographically and ecologically diverse samples were screened for sialic acid catabolism (Supplementary Table S1). These metagenomes represent about 1.0 × 10^12^ nucleotides of sequence data and 7.8 × 10^8^ contigs longer than 500 bp, including 1,380 from the Integrated Microbial Genomes and Microbiomes (IMG/M) Data Consortium, 1,132 from Human Microbiome Project (HMP), and 9 from Tara Oceans project. As the presence of *nanE* effectively represents the *nan* gene cluster (Li and Huang, 2022), we first detected the distribution of *nanE* in different environments. A total of 11,943 *nanE* genes were identified from human (*n* = 8,349, 69.9% of the total *nanE* genes), environmental (*n* = 2,534, 21.3%), engineered (*n* = 590, 4.9%), and non-human host-associated (*n* = 470, 3.9%) ecosystems, and were predicted in 1,890 metagenomes from 94.4% (1,069/1,132), 57.3% (625/1,090), 73.0% (103/141), and 58.9% (93/158) of each ecosystem, respectively.

**Figure 1:**
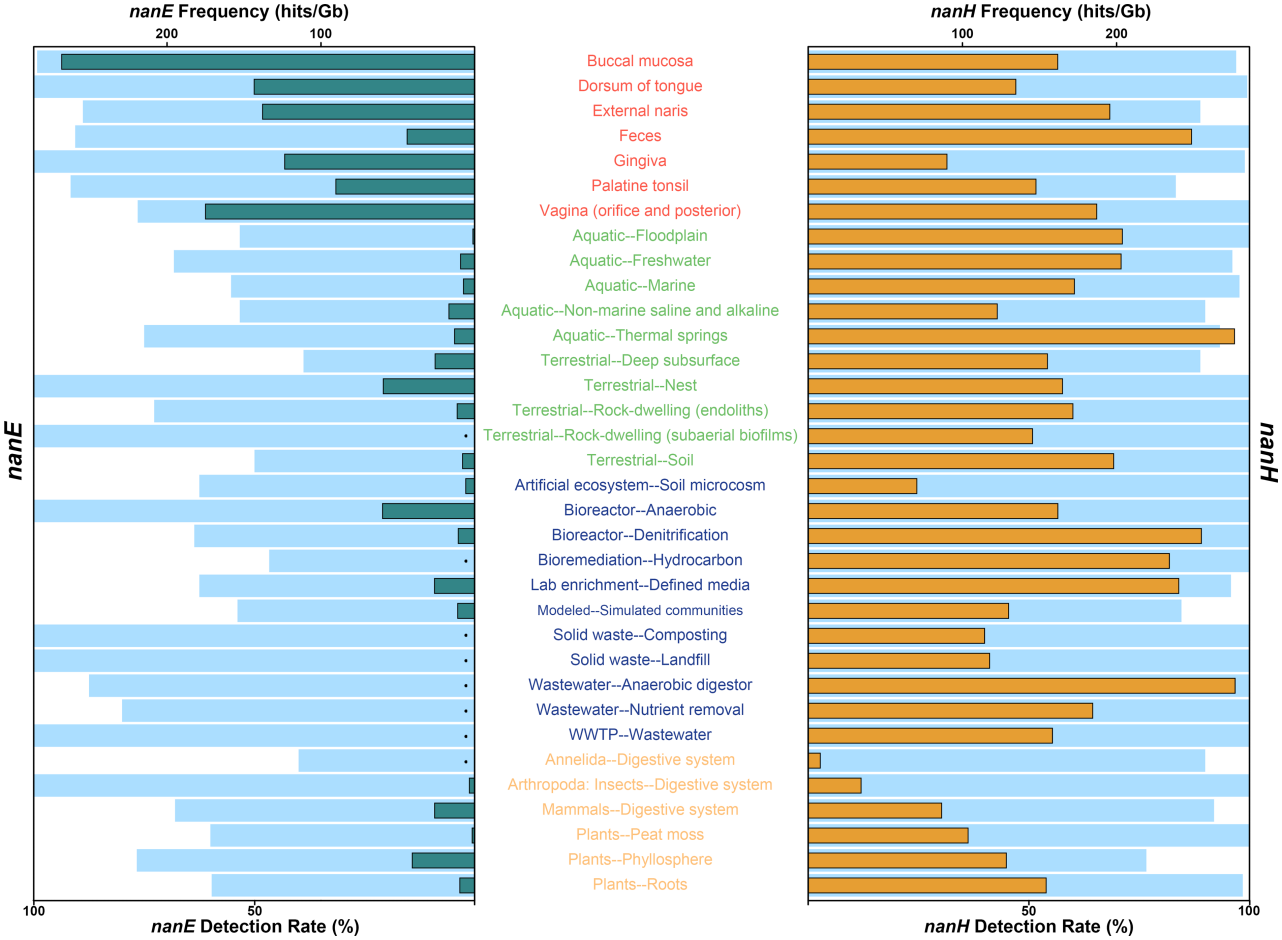
Detection rate and frequency of NanE (left) and sialidase NanH (right) in different environment types. For each ecosystem type or human body part, light blue bars indicate detection rate, and green and orange bars represent the average frequencies of NanE (left) and sialidase NanH (right) genes identified in metagenomes. Detection rate was calculated only when there were at least three metagenomes from each ecosystem type or human body part, and the average frequency was calculated only when both the total number of metagenomes and the NanE or sialidase genes in each metagenome were at least three. For clarity, only human body parts or ecosystem types contained at least three metagenomes with detected NanE and NanH genes, and with valid data for both *nanE* and *nanH* frequencies (hits/Gb), were shown. Detailed information can be found in Supplementary Table S2. Black dots, frequency not calculated. Texts are color coded by ecosystem: red, human body; green, environmental; blue, engineered; orange, non-human host-associated.

On a more granular level (Figure 1; Supplementary Table S2), across all human body parts, almost all (>99%) of metagenomes from buccal mucosa, dorsum of tongue, and gingiva contained predicted *nanE*, while the detection rates for others were around 90%, with the only exception of vagina (76.5%) and retroauricular crease (75.0%). Within environmental ecosystem, all of the nest and subaerial biofilms of rock-dwelling communities contained predicted *nanE*, followed by thermal spring (75.0%) and endoliths (72.7%); the detection rates of freshwater (68.3%), cave (66.7%), marine (55.3%), aquatic floodplain (53.3%), non-marine saline and alkaline (53.3%), and soil (50%) communities were no less than half; while the remaining five communities (oil reservoir, terrestrial deep subsurface, outdoor air, plant litter, and terrestrial floodplain) showed relatively low detection rates (< 40%). In addition, high rates of *nanE* have also been measured in engineered bacteria communities, with only bioremediation and built environments containing a comparatively small amount (but still greater than 30%). Meanwhile, we found that *nanE* existed in other non-human host-associated habitats, including digestive systems of insects (100%), annelids (40.0%) and other mammals (68.0%), as well as in phyllosphere (76.7%), peat moss (60.0%), and plant root (59.7%), but we did not find *nanE* in wood.

Although widely distributed, the frequency of *nanE* varies a lot in different habitats (Figure 1; Supplementary Table S2). As expected, metagenomes from human bodies had a roughly eight times higher frequency of *nanE* than those from other ecosystems. The highest frequency of *nanE* occurred in buccal mucosa (268.2 hits/Gb on average, similarly hereinafter), followed by vagina (174.9 hits/Gb), although the detection rate of *nanE* in vagina was only 76.5%. Next are communities from dorsum of tongue (143.3 hits/Gb), external naris (137.9 hits/Gb), gingiva (123.5 hits/Gb), and palatine tonsil (90.3 hits/Gb); while feces (44.1 hits/Gb) had the lowest frequency within human bodies. For non-human environments, communities of anaerobic bioreactor (60.0 hits/Gb), terrestrial nest (59.6 hits/Gb), and phyllosphere (40.8 hits/Gb) were the three most abundant, while the frequencies in typical free-living habitats (soil, freshwater, and marine) were lower than 10 hits/Gb. Interestingly, terrestrial deep subsurface (e.g., shale gas reservoir; 25.9 hits/Gb), non-marine saline, and alkaline water (17.0 hits/Gb), and thermal spring (13.3 hits/Gb) showed a relatively higher frequency.

**Figure 2:**
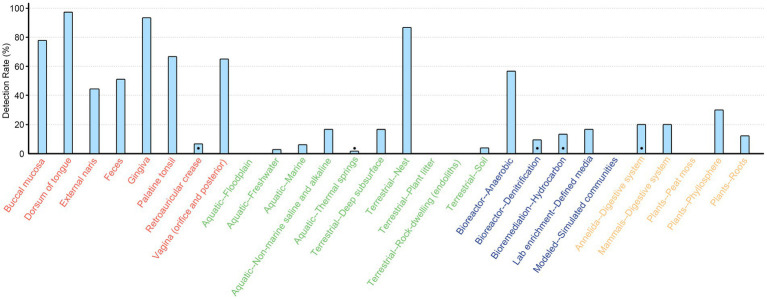
Detection rate of SAC gene cluster across different ecosystems. The rate was calculated only when there were at least ten metagenomes from each ecosystem type or human body part. Black dots indicate the gene clusters were only detected in less than three metagenomes. Texts are color coded by ecosystem: red, human body; green, environmental; blue, engineered; orange, non-human host-associated.

We then screened the contigs/scaffolds to detect whether the essential genes of SAC (*nanA*, *nanK,* and *nanE*) were colocalized within 10 open reading frames (ORFs) of each other forming a coexpression and regulation cluster. With this approach, a total of 1,988 SAC clusters were identified, comprising 16.6% of *nanE* genes. More than 90% (*n* = 1,816) were derived from human body parts, with 172 clusters found in environmental (*n* = 72), engineered (*n* = 53), and non-human host-associated (*n* = 47) habitats, showing a wide distribution in different kinds of ecosystems (Figure 2). Besides these, there were also 615 and 1,789 potential partial SAC clusters that only contains *nanE* and either *nanA* or *nanK* genes, respectively. However, 68.4% (*n* = 1,644) of these were located less than 10 ORFs from the contig or scaffold boundaries.

### Environmental distribution of predicted sialidase *nanH*

In general, sialidase genes (*nanH*) also showed a broad dispersal (Figure 1; Supplementary Table S2), and were significantly more prevalent than *nanE* across non-human environments (about 21.4 times on average; Wilcoxon test, *p* < 0.001), while they varied in different human body sites, with bacteria communities from dorsum of tongue, gingiva, and buccal mucosa containing less predicted *nanH* than *nanE*, but more in others. Specifically, the frequency of *nanH* was only about three fifths of that of *nanE* in buccal mucosa, but was 5.6 times in feces. Among free-living habitats, the highest frequency of *nanH* occurred in thermal springs (276.5 hits/Gb on average), even higher than in any sites of human bodies. In addition, metagenomes from floodplain, freshwater, and soil also contained high frequency at about 200 hits/Gb, and in floodplain, frequency of *nanH* was about 143 times higher than *nanE*. Some engineered habitats (e.g., anaerobic digestor, denitrification bioreactor, hydrocarbon bioremediation, and et al.) also had a high frequency of *nanH*. The lowest frequency of *nanH* was shown in the digestive system of insects and annelids.

### Taxonomic affiliation of *nanE* and SAC clusters

By using MEGAN with GTDB-tk taxonomy, we first determined the taxonomic distribution of *nanE* at different ranks. 98.3% (11,745/11,943) of the sequences were derived from bacteria, most of which could be assigned into known taxonomic categories at the higher levels (phylum, 82.0%; class, 81.0%; order, 74.2%; family, 69.2%). However, only 51.4% (6,138/11,943) and 5.5% (661/11,943) could be classified as known genera and species, respectively. In addition, about 25% of the *nanE* genes from non-human environments showed low homology (with amino acid identity of <70%) with any known genes in the NR database (Supplementary Figure S1). However, nearly all (98.47%) *nanE* genes in human bodies showed a much higher homology (with amino acid identity of >90%).

**Figure 3:**
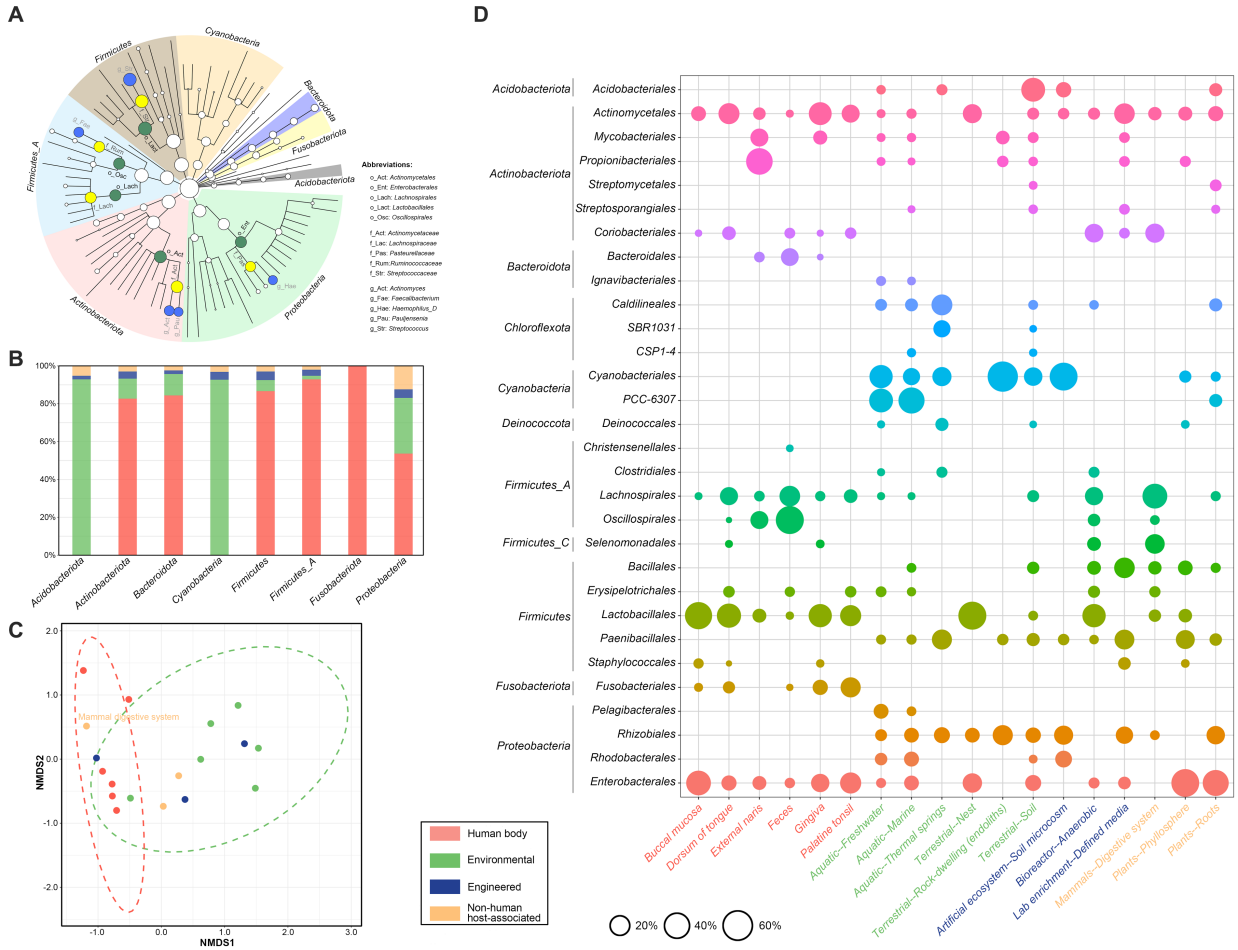
Taxonomic affiliation of NanE across different ecosystems. **(A)** Cladogram indicating the distribution of bacterial taxa containing NanE. The phylum, class, order, family, and genus levels are listed in order from inside to outside of the cladogram. The diameter of the circle is proportional to the relative abundance. Dominant phyla are shown by different background colors. Nodes of dominate taxa are marked by abbreviations and with green, yellow, and blue colors for levels of order, family, and genus, respectively. **(B)** Histogram of the ecological distribution of NanE in different phylum. **(C)** Non-metric multidimensional scaling (NMDS) analysis based on NanE taxonomic profile at order level. The analysis was conducted using Bray-Curtis distance. **(D)** Bubble plot shows bacterial orders that are differentially abundant among ecosystem types and human body parts. The size of circles represents the relative abundance. Only ecosystem types and human body parts with at least 30 and orders with at least 5 NanE proteins are considered. Different ecosystems are marked according to the color legend.

As shown in Figure 3A, the dominant phyla were *Firmicutes* (*n* = 2,522, 21.5%), *‘Firmicutes_A’* (*n* = 2,170, 18.5%), *Actinobacteriota* (*n* = 2,012, 17.1%) and *Proteobacteria* (*n* = 1,458, 12.4%), and the dominant orders included *Lactobacillales* (*n* = 1,946, 16.6%) and *Actinomycetales* (*n* = 1,311, 11.2%), followed by *Oscillospirales* (*n* = 1,061, 9.0%), *Enterobacterales* (*n* = 1,024, 8.7%), and *Lachnospirales* (*n* = 1,005, 8.6%). The dominant families included *Streptococcaceae*, *Actinomycetaceae*, *Lachnospiraceae*, *Ruminococcaceae,* and *Pasteurellaceae*, together amounting to nearly half (46.5%, 5,460/11,745) of the total sequences. Further, at the rank of genus, the five most abundant genera included *Streptococcus*, *Actinomyces*, *Faecalibacterium*, ‘*Haemophilus_D’,* and *Pauljensenia*. Finally, within 661 *nanE* genes from known species, 118 (17.9%) were from non-human environments; among these, *Pelagibacter* sp001438335 (*n* = 24), *‘Synechococcus_D lacustris’* (*n* = 20), and *‘Rhodobacter_B’* sp002701395 (*n* = 19) were the top three species, and all these genes were derived from lake, river, wetland, marine, or saltwater from algal raceway.

In addition, the majority of *nanE* encoded by *Acidobacteriota* and *Cyanobacteria* were from environmental habitats (Figure 3B), while by the other phyla in human bodies, except for *Proteobacteria* which showed a roughly even distribution in human and non-human environments. Taxonomic composition of *nanE* also varies dramatically between granular habitats (Figures 3C,D). For example, *nanE* of *Lachnospirales* and *Oscillospirales* were more abundant in feces or digestive systems, while *nanE* of *Propionibacteriales* presented more in external naris and were also found in low abundance in free-living habitats (e.g., freshwater, marine, rock, soil, and phyllosphere); members belonging to *Rhizobiales* were widely distributed in many kinds of environmental samples, but absent in human bodies. On the other hand, there were also some *nanE* genes from different bacterial groups that presented in many kinds of habitats, such as *Actinomycetales* and *Enterobacterales*.

We also checked the taxonomic affiliation of the contigs/scaffolds that contain the SAC clusters (Supplementary Table S3). Most SAC clusters (89.2%, 1,773/1,988) were on contigs/scaffolds affiliated with 9 bacterial phyla. Only 45.1% (878/1,988) could be classified at the genus level, with some representation from *Faecalibacterium* (20.6%, 181/878), *Actinomyces* (20.3%, 178/878), and *Streptococcus* (15.9%, 140/878). Only 32 clusters could be assigned at species level, the majority of which were derived from human bodies, except for a cluster of *Rhodococcus qingshengii* from enriched cells from switchgrass and soil in fabricated ecosystem (EcoFAB) chamber. Furthermore, a total of 34 clusters showed different taxonomy between *nanE* genes and corresponding contigs/scaffolds >10,000 bp in length, including 20, 9, and 5 clusters at order, family, and genus levels, respectively.

### Phylogeny of *nanE* and SAC clusters

**Figure 4:**
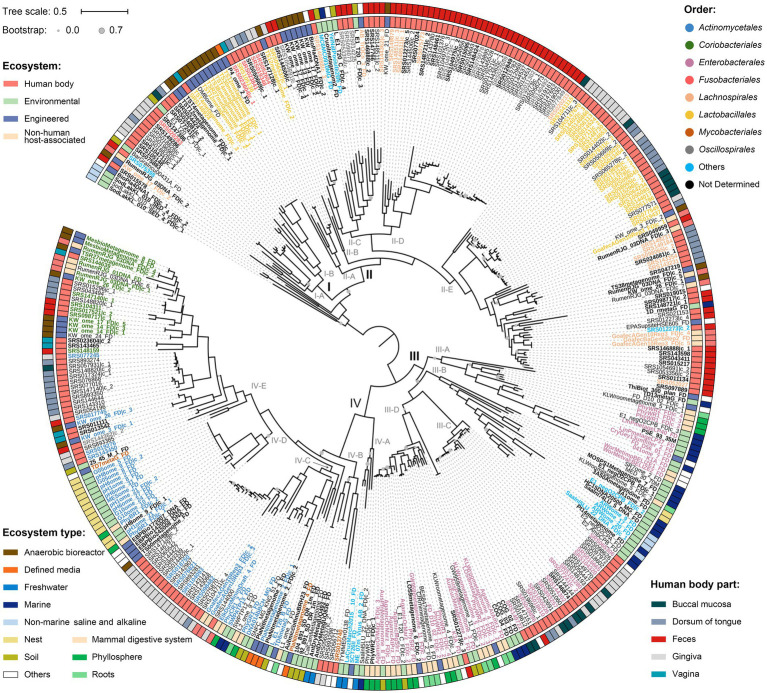
Phylogeny of the concatenated NanA-K-E proteins. The tree was produced using FastTree. Clusters from human body parts are filtered by CD-HIT with threshold 0.97 to remove highly similar sequences. Nodes with bootstrap values less than 70% are marked with grey circles in different sizes. “I” to “IV” indicate genetic lineages, and “A” to “E” indicate clades. Scale bar indicates 50% sequence divergence. Inner and outer circles indicate the source of the SAC gene clusters at ecosystem and ecosystem type/human body part levels, respectively. Colored labels of the SAC gene clusters indicate different orders, and bold fonts indicate the gene clusters are located in contigs/scaffolds longer than 10,000 bp. SAC gene clusters from human body parts are filtered by CD-HIT with threshold 0.97 to remove highly similar sequences. Details of the gene clusters are listed in Supplementary Table S3.

All protein sequences of *nanE* were firstly used to reconstruct a phylogeny of environmental diversity (Supplementary Figure S2). Overall, the *nanE* derived from human bodies, environmental, engineering, and host-associated habitats showed an interspersed distribution in the tree, indicating the occurrence of large amounts of gene transitions between habitats. Next, we concatenated proteins encoded by the clustered *nanA/K/E* genes to obtain a more precise phylogenetic tree (Supplementary Table S3). We created a sequence alignment for a total of 371 SAC clusters after removal of 1,617 redundant ones derived from human bodies. A midpoint-rooted phylogeny showed four major lineages with high ecological diversities (Figure 4). When focusing only on the non-engineered habitats, lineages I and II mainly contained clusters from human bodies and mammalian digestive systems, while clusters from nearly all kinds of ecosystems mixed in lineages III and IV. Specifically, lineage I contained 27 clusters, more than half of which were from mammalian hosts, with only two from soil inserted in clade I-A and clade I-B. In lineage II, 85.9% clusters were from mammalian hosts distributed in all five clades, while clades II-C and II-E also contained 6 from soil, marine, and thermal spring. Lineage III contained mainly free-living and plant-associated derived SAC clusters, except for a monophyletic subgroup in clade III-C with 24 mammal-sourced clusters. Lineage IV could be further divided into five clades; clusters in clades IV-A and IV-D were represented by nest and aquatic habitats, clade IV-E by mammal hosts, while clades IV-B and IV-C contained clusters from both host and free-living habitats, although clusters from similar ecosystems also tend to form individual subgroups within each clade. In addition, SAC clusters from different kinds of engineered environments also showed habitat correlations. For example, clusters from anaerobic bioreactor were more likely to gather with those from mammal hosts, while clusters from waste water, soil microcosm, irrigation canal, and environmental bioremediation habitats were more likely to group with those from free-living or plant-associated habitats. Generally, SAC clusters from the same habitats tend to group together in shallow branches, although they could also be clustered in different lineages, and interchanges may also happen frequently between different habitats.

Furthermore, in this NanA-K-E tree, although most SAC clusters from the same orders tended to cluster together, taxa from the same order could also distribute in different lineages or clades. For instance, SAC clusters of the orders *Fusobacteriales* and *Lactobacillales* were located in both lineages I and II, and clusters from order *Mycobacteriales* presented in both clades IV-B and IV-E. Meanwhile, some shallow clades contained SAC clusters from various orders, such as clade II-C, which contained 22 clusters from at least four different orders or two different classes.

## Discussion

Sialic acids have been reported to mainly localize on the surface of cell membranes with multiple cell biological functions in the physiology of humans and other metazoan animals (Schauer and Kamerling, 2018). Microbial SAC is one of the processes critical for mammal pathogenesis, thus, it has mainly been studied in commensals and pathogens. By deep sequence mining of a diverse and extensive metagenome dataset, in this study we gave a reliable picture of the global occurrence and diversity of putative microbial SAC in different human body parts, and different types of habitats and taxa. The results suggested that bacterial SAC widely exists not only in commensals and pathogens in mammals, but also in other common environments, such as free-living, plant-associated, and certain kinds of engineered habitats. These SAC clusters in different habitats or taxa might experience multiple and different evolutionary processes, and HGT events might play important roles in the spread of the clusters.

### Ecology and distribution of SAC-containing bacteria

#### Human and vertebrate microbiomes

As expected, microbial SAC genes were highly prevalent in the human body, suggesting a potential disease risk. The frequency of SAC was the highest in buccal mucosa. This is in accordance with its nomenclature, as sialic acid was initially described in saliva and thus received its name (Vimr et al., 2004). The next was in vagina; vaginal sialidase activity and high levels of free/liberated sialic acid are diagnostic of bacterial vaginosis (BV), as improved growth or colonization by sialic acid consumers can lead to sialoglycan depletion and the degradation of protective vaginal mucus barriers (Lewis et al., 2013; Agarwal and Lewis, 2021). Interestingly, among all body parts, the feces harbored both the highest frequency of sialidase genes and the lowest frequency of SAC degrading genes, perhaps suggesting that in the human gut, only a small fraction of bacterial commensals and pathogens could derive nutritional benefits from host sialoglycans where exogenous sialidases were produced by themselves or other bacteria. This phenomenon also existed in other mammal digestive systems (e.g., cow, elk, goat). Perhaps this kind of cooperation contributes to the overall sialic acid degradation capability of gut communities (Coker et al., 2021). Further efforts should be made to evaluate the sialic acid concentrations in different body parts to assess whether the prevalence and frequency of microbial SAC is consistent with this.

#### Free-living habitats

To date, very few studies have shown that free-living microbes can also utilize sialic acids (Gruteser et al., 2012; Li et al., 2019; Tomás-Martínez et al., 2022). Here, we demonstrated that microbial sialidase activity was present in almost all samples from environmental ecosystems with high frequency, and sialic acid degrading capacity was also present in more than half of the samples (e.g., soil, marine, freshwater, rock), although the frequency was much lower than that in human bodies. Particularly, the highest frequency of sialidases was detected in thermal spring metagenomes, suggesting a previous underestimate of their abundance and diversity. A recent functional metagenomic screening of thermal spring has uncovered a new GH156 sialidase family, and further study has confirmed that these GH156s are frequently encoded in human microbiome (Chuzel et al., 2018; Mann et al., 2022). Furthermore, engineered systems, which generally reflect the richness of restricted species, contained microbial SAC and displayed a relatively high phylogenetic diversity. All these above perhaps suggested a wide distribution of the microbial SAC, rather than one limited in higher animal hosts.

#### Insect and plant associated habitats

It was known that sialic acid content in insects is low and totally lacking in plants (Ghosh, 2020). Our recent studies on *Streptomyces* species have shown that SAC is significantly associated with free-living strains, and the insect-associated group likely lost the gene clusters to maintain harmonious coexistence with their hosts (Li et al., 2019; Wang et al., 2022). However, our screen here has uncovered the presence of microbial SAC in some insect-associated habitats, for example, ant gut and dump. As sialic acid and sialylation play important roles during insect development (Roth et al., 1992; Koles et al., 2009), these SAC carriers perhaps should be treated as pathogens for insects. In addition, although sialic acids have not been reported from phylum *Annelida* (Warren, 1963; Ghosh, 2018), we have detected *nanE* genes in metagenomes of marine gutless worm symbionts. Furthermore, metagenomes from plant-associated environments, including peat mosses, roots, and phyllosphere, were also positive for microbial SAC.

### Potential existence of microbial SAC in novel organisms

Currently, as a commonly held view that sialic acid is regularly present in higher animals, our understanding of microbial SAC mainly comes from pathogenic and commensal bacteria of mammals (Severi et al., 2007; Olson et al., 2013; McDonald et al., 2016). Recently, our phylum-level investigation, which based on genome sequences derived from pure culture of actinobacteria, indicated that SAC also exists in many free-living bacterial species. Using culture-independent metagenomic data, in this study we have largely expanded our current understanding of the distribution and taxonomic diversity of microbial SAC, since about half of the *nanE* genes and SAC gene clusters identified could not be classified beyond genus level. In addition, along with the low similarity values against the known proteins in database, our results indicate a greatly underestimated resource for microbial SAC in non-human habitats, and even that these microbes should be reconsidered as potential pathogenic threats to human health. For example, we have detected the SAC gene clusters from members of *Rhizobiales* (genus unknown) in phycosphere, and also *nanE* genes in other free-living or plant-associated habitats, but did not detect these in human bodies. It has been reported that the order *Rhizobiales* contains nitrogen-fixing bacteria that live in a symbiotic relationship with plant roots, and also human pathogenic strains (Berman, 2012), perhaps where SAC may confer virulence. Similar phenomenon was also shown in *Rhodobacterales*. Particularly, we have detected a high abundance of *nanE* genes in *Cyanobacteriales*, *Caldilineales*, *Ignavibacteriales* and *Paenibacillales*, without any SAC gene cluster. Indeed, some cyanobacteria have been shown to encode enzymes for sialic acid biosynthesis and also the activity to convert ManNAc to GlcNAc (Lee et al., 2007; Lewis et al., 2009), but until now, to our knowledge there was no evidence that strains of cyanbacteria could utilize sialic acids. Further experiments are needed to verify the potential existence/function of SAC in these organisms.

### Potential physiological and ecological roles of microbial SAC

Although long been considered as virulence determinant, microbial sialidases and SAC may have beneficial effects on humans (Ley, 2014; Keil et al., 2022). In this study, we have detected several SAC clusters in species traditionally considered to be probiotic bacterium. For example, SAC gene clusters have been detected in *Faecalibacterium prausnitzii*, an indicator of intestinal health widely existing in all animals (Miquel et al., 2013), and in *Corynebacterium matruchotii*, an inhabitant of the oral cavity of humans and animals which is rarely associated with human disease (Kim and Reboli, 2015). However, the ability to catabolize sialic acids in either of the two species has not been reported yet. A better understanding of the distribution of SAC in different organisms, as well as in different body parts, will help shed light on the clarification of the pathological and physiological processes, and further facilitate the development of effective treatments for bacterial infection.

However, the ecological roles of the SAC in non-mammal-associated environments were still poorly understood. For example, while plants themselves did not express sialic acids, sialic acid-binding lectins that specifically recognize sialic acid residues have been reported to occur in plants with diverse specificity (Lehmann et al., 2006). Microbial SAC may provide plants with a defensive mechanism with a means of recognizing and combating sialylated pathogens and/or predators (Schauer and Kamerling, 2018). In addition, soil-dwelling bacterium with the ability of SAC may utilize sialic acids, perhaps from surrounding fungi or decomposing animals (Gruteser et al., 2012), to survive adverse conditions and outcompete other soil dwellers. Nevertheless, further efforts are needed to determine the ecological roles of the SAC-positive strains in these overlooked environments (e.g., pure/enrichment culture or microcosm techniques).

### Implications of evolution of microbial SAC

The results of the present study reinforced our previous finding that HGT plays a significant role in the evolution and distribution of microbiol SAC (Li and Huang, 2022). We have shown abundant intersections between *nanE* genes and the gene clusters from different taxon or habitats in phylogenetic trees. Also, discrepancies between the taxonomic affiliation of *nanE* genes versus their corresponding contigs/scaffolds were also detected. All these findings suggested that the microbiol SAC has undergone extensive horizontal transfer among different groups of microorganisms and habitats. Despite these, the mammal-associated microbial SAC gene clusters were spread equally across all the lineages. However, about three-fourth (75.9%, 120/158) of the non-mammal-associated clusters were located in linages III and IV, suggesting a special environmental pressure and a relatively independent evolution history. It seems that clusters in linages I and II were not well suited to that free-living or plant-associated lifestyles, and their sporadic appearance in non-mammal-host habitats may mainly come from occasional HGT events. Similarly, the phylogenetic relationship of clade III-C (human-sourced SAC gene clusters forming a monophyletic branch in lineage III), and the location of cluster SRS012273|c_3 in clade IV-D (Figure 4), probably show a ‘reverse-transfer’ of the SAC clusters from free-living to host-associated habitats. These findings probably mean that some free-living bacteria have evolved an adaptive ability to utilize sialic acids efficiently as nutrient sources to cope with adverse conditions, rather than a random acquisition that happens over and over again.

### Study limitations

Our study has some limitations. First, our study was based on the publicly available metagenomes from diverse environments and databases, therefore the potential biases, especially between human bodies and free-living habitats, on sequencing depths, quality filtering, and assembly methods could impact the results. Actually, by comparing the detection rate and frequency of *nanE* genes in microbiome between human feces (from HMP) and mammal digestive systems (from IMG/M Data Consortium) (90.6% vs. 68.0%; 44.1 vs. 26.3 hist/Gb), combined with the NMDS plot which showed an obvious clustering of these genes, we hold the opinion that the results were not strongly affected. In addition, we did not find a linear relationship between metagenome size and the number of predicted *nanE* genes (*R^2^* = 0.002). However, a strong linear relationship was found for *nanH* genes (*R*^2^ = 0.736), suggesting a high frequency and unexplored diversity of *nanH* genes in every microbiome, although the deviations among samples could arise.

Another potential limitation is how extent the presence of *nanE* genes could represent the ability of SAC. Indeed, we have shown in phylum *Actinobacteria* that *nanE* could mainly represent the SAC gene cluster (Li and Huang, 2022), there still may be exceptions in some other microorganisms. Although we have detected the SAC at gene cluster level, more SAC-positive isolates should be included in future studies. However, although only containing parts of the SAC pathway, these microorganisms might have their own way to affect the whole microbial community (Coker et al., 2021). Besides, as there were amounts of partial SAC clusters falling at contig/scaffold boundaries in the assembly, the actual level of distribution and diversity of SAC clusters could still be underestimated. Despite these, functional assays, especially for isolation of SAC-containing bacteria from non-host-associated habitats and transcriptome-based survey of the gene cluster, were needed to give more convincing evidences for the global distribution of microbial SAC.

Nevertheless, our findings have revealed the global distribution and evolution patterns of putative microbial SAC, and have significantly expanded its recognized diversity. This challenges the prevailing notion that microbial SAC is mainly restricted to pathogenic and commensal bacteria in mammals, and will help to provide the backbone for further studies on its ecological roles and potential pathogenesis, especially in free-living and plant-associated ecosystems.

## Materials and methods

### Collection of pre-assembled metagenomes

We aggregated publicly available data from three main sources, including pre-assembled metagenomes and raw shotgun sequencing read data (June 2022). Pre-assembled metagenomes (*n* = 2,512) were downloaded from Integrated Microbial Genomes and Microbiomes (IMG/M) (*n* = 1,380)^1^ and Human Microbiome Project (HMP) (*n* = 1,132).^2^ For raw shotgun sequencing read data, nine randomly selected samples from *Tara* Oceans metagenomes from different locations were used to reconstruct the assembled contigs according to the workflow outlined by Delmont et al. (2018). Briefly, raw data was filtered using the illumine-utils library (Eren et al., 2013) and assembled using MEGAHIT with a minimum contig length of 500 bp (Li et al., 2015). For all these metagenomes (*n* = 2,521), contigs shorter than 500 bp in length were discarded. We then used MetaQUAST to assess the assembly quality (Mikheenko et al., 2016), requesting all these metagenomes had N50 values higher than 1,000 bp, a genome size larger than 10 M bp, and the number of N’s per 100 kbp lower than 100 bp. Metagenome sources were obtained and classified according to their metadata available. Specifically, the non-human sourced metagenomes were classified using the GOLD five-level ecosystem classification system (Mukherjee et al., 2019). A summary of the metagenomes used in this study and their classifications based on the types of environments and samples is provided in Supplementary Table S1.

### Identification and taxonomic affiliation of SAC genes and gene clusters

All the protein coding sequences (CDSs) were called from scaffolds/contigs using Prodigal with “meta” mode (Hyatt et al., 2010). In order to identify putative SAC genes (including *nanA*, *nanK*, *nanE*, and *nanH* genes), we used the pipelines described in our previous study (Li and Huang, 2022). Briefly, all CDSs were compared against the PFAM-A database (version 35.0) to identify those containing conserved protein domains of SAC, using HMMER package (Eddy, 2011) with a maximum E-value of 1e-5, and the resulting protein sequences were then used as queries in a Reverse Position Specific-BLAST (RPS-BLAST) search (E-value <1e-5 and coverage >70%) against NCBI’s Conserved Domain Database (CDD) (Marchler-Bauer et al., 2015) to confirm the presence of SAC-associated domains. Finally, to avoid false positive annotation, the resulting NanE sequences were further checked by MEGAN (Huson et al., 2016) for functional classification by comparison with COG, SEED, and INTERPRO databases. Gene frequency was calculated according to the number of gene hits found for every 1 Gbp. Only if the *nanA/K/E* genes in a single contig/scaffold were within 10 open reading frames (ORFs) of each other, we determined the presence of SAC gene cluster (Almagro-Moreno and Boyd, 2009).

MEGAN was also used to explore the taxonomic diversity of SAC genes and gene clusters. Firstly, all NanE sequences were used to conduct a diamond blastp (Buchfink et al., 2015) search against NCBI’s non-redundant database. The top-ten best hits for each protein sequence were used for taxonomic rank assignments based on the lowest common ancestor algorithm with GTDB taxonomy (Chaumeil et al., 2019). Similarly, for the SAC gene cluster, diamond blastx result was used as input to determine the taxonomic assignment for each encoding contig/scaffold with a maximum of 50 best hits.

### Phylogenetic analyses

Multiple protein sequence alignments of each gene were performed using MAFFT (Katoh and Standley, 2013) and trimmed with TrimAl (Capella-Gutierrez et al., 2009). The NanE phylogenetic tree was built by using the software FastTree with the LG amino acid substitution model (Price et al., 2010). For the SAC gene cluster phylogeny, trimmed protein sequences of clustered *nanA/K/E* genes (within 10 ORFs of each other in the same contig or scaffolds) were concatenated. CD-HIT algorithm (Fu et al., 2012) was implemented to remove the sequence redundancy from human sources with threshold 0.99 for NanE phylogeny and 0.97 for SAC gene cluster phylogeny. Phylogenetic trees were rendered and visualized with iTOL (Interactive Tree Of Life) online tool^4^ (Letunic and Bork, 2021).

## Data availability statement

All assembled metagenomes analyzed in this study can be accessed through the assembly IDs from Integrated Microbial Genomes and Microbiomes (IMG/M) Data Consortium and Human Microbiome Project (HMP). Metagenomic reads from the *Tara* Oceans project are publicly available at European Bioinformatics Institute with project number ERP001736. Details of the assembly IDs and metadata are listed in Supplementary Table S1.

## Author contributions

YL: Conceptualization, Data curation, Formal analysis, Funding acquisition, Investigation, Methodology, Project administration, Resources, Software, Supervision, Validation, Visualization, Writing – original draft, Writing – review & editing. YF: Data curation, Software, Validation, Visualization, Writing – review & editing. XM: Validation, Writing – review & editing. YW: Validation, Writing – review & editing. JL: Conceptualization, Funding acquisition, Project administration, Supervision, Validation, Writing – review & editing, Data curation.

## Footnotes

^1^ https://img.jgi.doe.gov

^2^ http://hmpdacc.org

^4^ http://itol.embl.de/itol.cgi
