# Supplementary material for: Metagenomic survey reveals global distribution and evolution of microbial sialic acid catabolism
Source: Front Microbiol. 2023 Sep 29;14:1267152. doi: 10.3389/fmicb.2023.1267152 (PMC10570557; doi:10.3389/fmicb.2023.1267152)
Supplement: Supplementary file 2 [file Image_1.PDF]

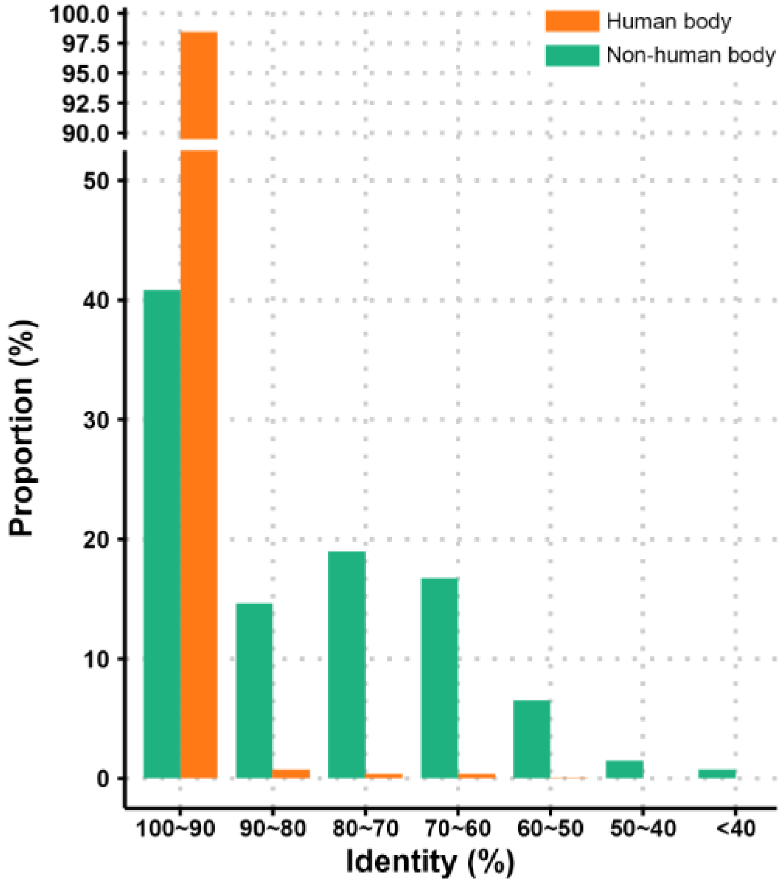

Figure S1. Distribution of amino acid identity of NanE against NR database. Diamond BLASTp was applied to search for homologous sequences of NanE in NCBI's NR database with E-value < 1e-5. Only the best/highest-scoring matches were considered.

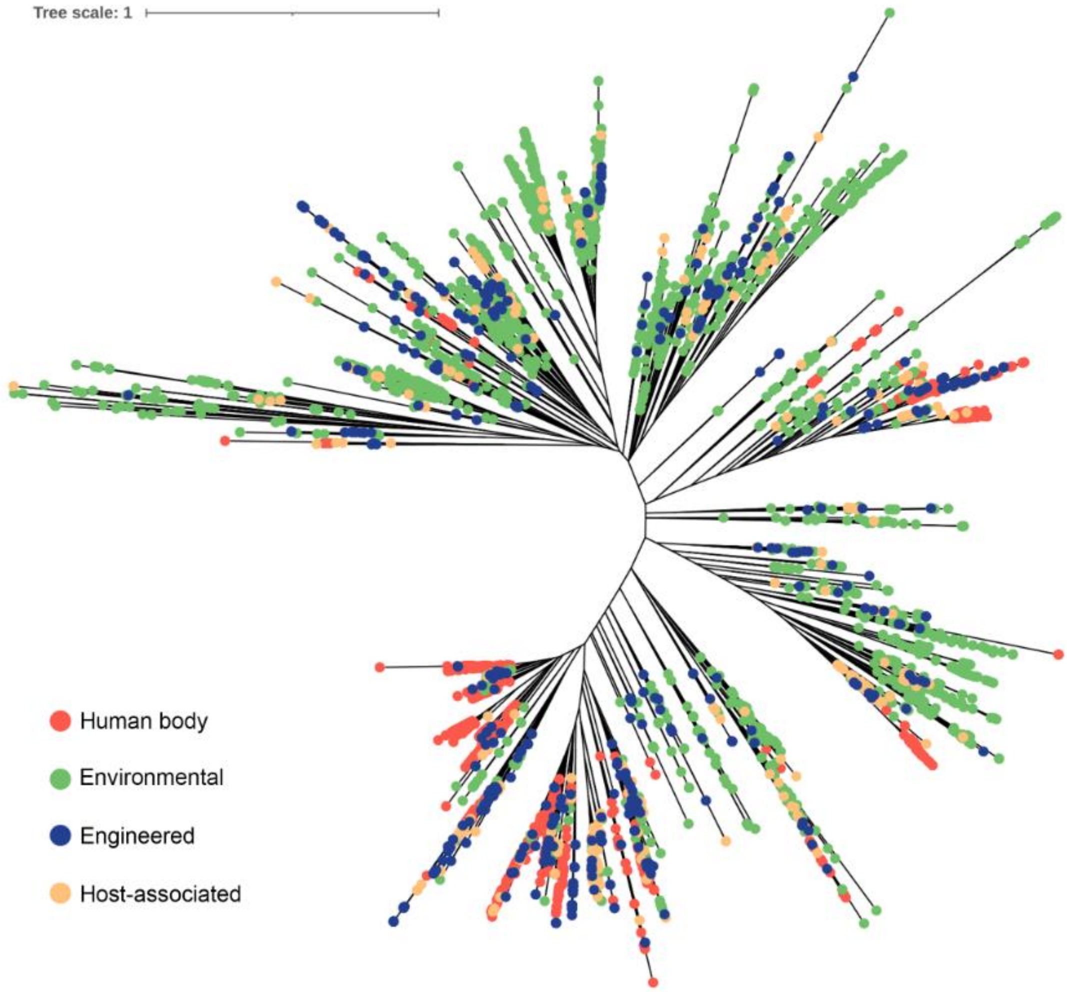

Figure S2. Phylogeny of the NanE proteins identified in all habitats. The tree was produced by FastTree software. Proteins collected from different ecosystems are marked as circles with different colors. For a better visualization, proteins from human bodies are filtered by CD-HIT with threshold 0.99 to remove highly similar sequences.
